# Singlet Fission in Lycopene H-Aggregates

**DOI:** 10.1021/acs.jpclett.3c02435

**Published:** 2023-10-27

**Authors:** William Barford

**Affiliations:** Department of Chemistry, Physical and Theoretical Chemistry Laboratory,University of Oxford, Oxford, OX1 3QZ, United Kingdom

## Abstract

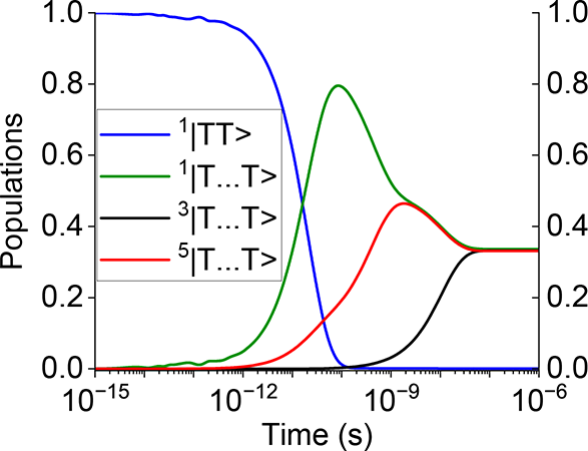

A theory of singlet fission (SF) in carotenoid dimers is applied to explain the SF in lycopene H-aggregates observed after high-energy photoexcitation. The explanation proposed here is that a high energy, delocalized bright ^1^*B*_*u*_^+^ state first relaxes and localizes onto a single lycopene monomer. The high-energy intramonomer state then undergoes internal conversion to the 1^1^*B*_*u*_^–^ state. Once populated, the 1^1^*B*_*u*_^–^ state allows exothermic bimolecular singlet fission, while its internal conversion to the 2^1^*A*_*g*_^–^ state is symmetry forbidden. The simulation of SF predicts that the intramonomer triplet-pair state undergoes almost complete population transfer to the intermonomer singlet-pair state within 100 ps. Simultaneously, ZFS interactions begin to partially populate the intermonomer quintet triplet-pair state up to ca. 2 ns, after which hyperfine interactions thermally equilibrate the triplet-pair states, thus forming free single triplets within 50 ns.

Singlet fission in polyacenes is a photophysical process that has been studied for over 50 years, with numerous reviews covering the topic.^1−5^ In that time, a consensus seems to have emerged as to the initial mechanisms of this process: namely, that after photoexcitation into the bright singlet state of a single chromophore, this state undergoes bimolecular fission into two triplets via a two-step electron–hole transfer.^2,6,7^ Research into singlet fission in polyacenes is now generally focused on how to improve the triplet yield in pursuit of technological applications, which requires a full understanding of the fate of the triplet-pair.^4^

In contrast, an understanding of singlet fission in oligoenes,^8,9^ polyenes^10−12^ and carotenoids^13−19^ is still in its infancy.^3,20−23^ A consensus does not exist on the fundamental question as to whether singlet fission in these systems proceeds in the same manner as for polyacenes (i.e., directly from a bright state),^16^ or whether an intermediate intramolecular correlated triplet-pair state is involved in the process.^24,25^

The possibility that an intermediate intramolecular triplet-pair state is involved in singlet fission is suggested by the well-known fact that the lowest excited singlet states of polyenes are indeed a superposition of correlated triplet-pairs^26−28^ and charge-transfer exciton states^28^ that are populated within 50 fs of photoexcitation of the “bright” Frenkel exciton state. However, since the triplets in the *lowest* energy “dark” state (i.e., the 2^1^*A*_*g*_ state) are strongly bound,^28,29^ a theory that involves *a* “dark” state in singlet fission also has to explain why it is an exothermic process with a high yield of free triplets.

In this Letter, we develop a theoretical model of singlet fission that qualitatively explains a recent experimental investigation of singlet fission in lycopene H-aggregates by Kundu and Dasgupta.^18^ In their work, Kundu and Dasgupta observed that singlet fission only occurs when the lycopene H-aggregates are excited at ca. 350 nm (ca. 3.5 eV), i.e., directly into the blue-shifted H-aggregate absorption band. In contrast, excitation in the 400–500 nm (2.5–3.1 eV) range, which corresponds to the monomer absorption band, does not cause singlet fission (see Figure 1 of ref (18)). This observation suggests that to facilitate singlet fission, excess energy is needed and/or that the monomers must be electronically coupled.

**Figure 1:**
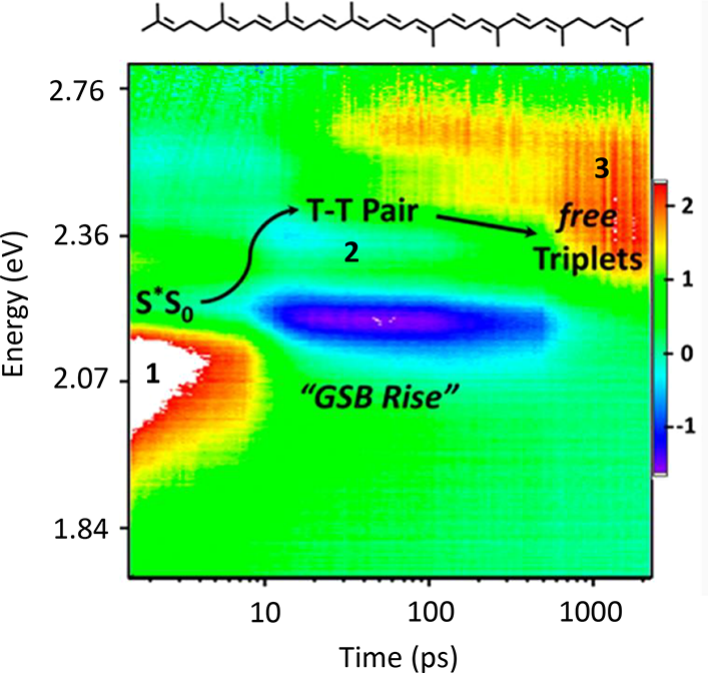
Measured^18^ transient excited-state-absorption of a lycopene H-aggregate following photoexcitation at 3.15 eV. See Figure 4 and the text following it for an explanation of the photophysics. Reproduced and modified from ref (18). Copyright 2021 American Chemical Society.

Another important observation of Kundu and Dasgupta is that the transient excited state absorption (ESA) reveals an intermediate state generated after photoexcitation that has the characteristic signature of a member of the “2*A*_*g*_”family of strongly correlated states. As has been shown by Barford and co-workers,^22,29^ it is the charge-transfer exciton component of these correlated states that absorbs at ca. 2.0 eV, at an energy red-shifted by 0.3 eV from the free-triplet signal. [See section 2 of the SI.] This is precisely what is observed by Kundu and Dasgupta, as shown in Figure 1. [In contrast, ESA from the “bright” *S*_2_ state is predicted to occur at ca. 1 eV.^29^] Importantly, the modeling of their transient ESA data led Kundu and Dasgupta to associate this feature as a higher-energy, intermediate triplet-pair state, i.e., 1^1^*B*_*u*_^–^ or *S*_1_^*^. This is significant, because as shown below, unlike for the 2^1^*A*_*g*_^–^ state, exothermic intermonomer singlet fission is possible from the 1^1^*B*_*u*_^–^ state.^29,30^

Using time-dependent DMRG calculations of the Hubbard-UV-Peierls model, Manawadu and co-workers^30,31^ simulated the internal conversion from the bright state in the related carotenoid, zeaxanthin. Zeaxanthin has 18 conjugated C atoms (i.e., 9 double bonds) and, like lycopene (shown in Figure 1), possesses *C*_2*h*_ symmetry. According to the simulation, excitation into the bright 1^1^*B*_*u*_^+^ state is followed within 10 fs by adiabatic internal conversion to the 1^1^*B*_*u*_^–^ state via an avoided crossing of *S*_2_ and *S*_3_. However, as a consequence of *C*_2*h*_ symmetry, to zeroth-order in the Born–Oppenheimer approximation, subsequent internal conversion to the 2^1^*A*_*g*_^–^ state (*S*_1_) is symmetry forbidden.

Here, we propose a somewhat different mechanism of internal conversion for a lycopene monomer within a H-aggregate. In particular, optical excitation into the blue-shifted absorption band of the H-aggregate excites a high-energy ^1^*B*_*u*_^+^ state that is delocalized over a number of monomers. As a consequence of electron–nuclear coupling and intermolecular interactions, this state rapidly relaxes and localizes onto a single lycopene monomer. Electronic localization onto a single monomer is facilitated by the large density of intramonomer electronic states at the aggregate absorption energy. The intramonomer excited state then undergoes nonadiabatic internal conversion to the intramonomer 1^1^*B*_*u*_^–^ state. (This process is similar to the nonadiabatic relaxation and localization of high energy excited states of a conjugated polymer onto a single chromophore described in ref (32).) The time scale for internal conversion is determined by how fast energy is dissipated, but it is expected to ca. 100 fs.^32^ Bimolecular exothermic singlet fission then follows, as the relaxed energy of the 1^1^*B*_*u*_^–^ state lies about 0.4 eV above the relaxed energies of a pair of triplets on separate monomers. Importantly, because of symmetry constraints, subsequent internal conversion from the 1^1^*B*_*u*_^–^ state to the 2^1^*A*_*g*_^–^ state is a slow Herzberg–Teller-allowed process. Conversely, bimolecular interstate conversion from 1^1^*B*_*u*_^–^ to *T*_1_^*i*^⊗*T*_1_^*j*^ (where *i* and *j* label the monomers) is not symmetry forbidden, provided that the monomers in the H-aggregate are not perfectly aligned. This scheme is shown schematically in Figure 2. [Alternatively, it has been proposed by Gierschner^33^ that the high-energy “bright” Frenkel exciton aggregate state might relax through the manifold of aggregate states, creating a low-energy “dark” Frenkel exciton aggregate state. While it is possible that such a process might lead to exothermic singlet fission, the ESA data^18^ does not appear to indicate a low-energy “dark” Frenkel exciton aggregate state, while it does indicate an intramolecular triplet-pair state.]

**Figure 2:**
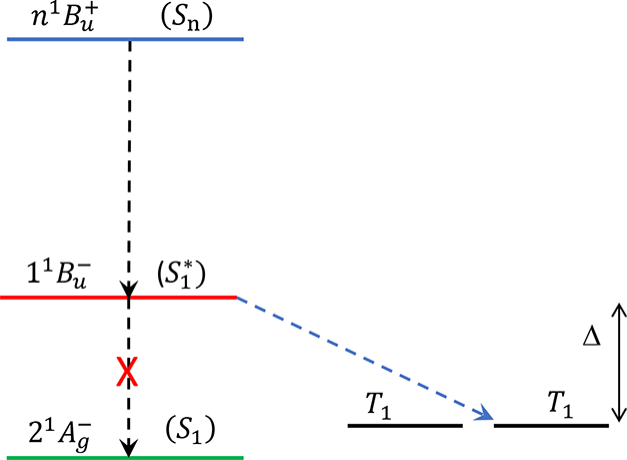
A schematic energy level diagram for some states of lycopene (the adiabatic state labels are in parentheses). The singlet states are on the left, while on the right is the energy level of two triplet states on separate monomers. Here we assume that there is localization and internal conversion from a high-energy, delocalized bright state (here labeled *S*_*n*_) into the intramonomer 1^1^*B*_*u*_^–^ state, which then undergoes exothermic intermonomer singlet fission. To zeroth-order in the Born–Oppenheimer approximation, interconversion from the 1^1^*B*_*u*_^–^ state to the 2^1^*A*_*g*_^–^ state is symmetry forbidden. Bimolecular interstate conversion from 1^1^*B*_*u*_^–^ to *T*_1_^*i*^⊗*T*_1_^*j*^ (where *i* and *j* label the monomers) is not symmetry forbidden provided that the monomers in the H-aggregate are not perfectly aligned. Δ is the exothermic driving energy from the 1^1^*B*_*u*_^–^ state.

Following the generation of intermonomer triplets, the triplets diffuse into the aggregate, thus preventing intermonomer recombination into the 2^1^*A*_*g*_^–^ state. Alternatively, as shown in ref (22), additional torsional relaxation can stabilize the free triplets relative to the 2^1^*A*_*g*_^–^ state, thus making recombination an endothermic process.

Before discussing our simulations of the singlet fission process, we first address the question as to why excitation into the monomer absorption band does not cause singlet fission in lycopene.^18^ One possible and obvious explanation is simply that absorption into the monomer band implies that the monomer is electronically decoupled from other monomers, and thus, a bimolecular fission mechanism is inoperable.

For another possible explanation, we turn to the energy levels of monomeric lycopene obtained via DMRG calculations^34^ of the Hubbard-UV-Peierls model for a monomer of 22 carbon sites. (Details of this model and its parametrization are given in the SI.) In both the theoretical and experimental literature, there is uncertainty about the exact relative energies of the vertical 1^1^*B*_*u*_^+^, 2^1^*A*_*g*_^–^, and 1^1^*B*_*u*_^–^ states in carotenoids. These energies are highly sensitive to monomer-length and dielectric screening. Some high-level ab initio calculations^35,36^ predict that the vertical 2^1^*A*_*g*_^–^ state lies above the vertical 1^1^*B*_*u*_^+^ state. In this case, excitation into the 1^1^*B*_*u*_^+^ state causes a 2^1^*A*_*g*_^–^ – 1^1^*B*_*u*_^+^ level crossing. Alternatively, some semiemprically parametrized Hamiltonians^30^ predict that the vertical 2^1^*A*_*g*_^–^ state lies below the vertical 1^1^*B*_*u*_^+^ state, while the vertical 1^1^*B*_*u*_^–^ lies above it. In this case, excitation into 1^1^*B*_*u*_^+^ causes a 1^1^*B*_*u*_^–^ – 1^1^*B*_*u*_^+^ level crossing. In previous work, we have modeled both situations by modifying the parameters of the Hubbard-UV model.^22^ The energy levels for lycopene for the two different semiempirical parameter sets are listed in Figure 3. Panel (a) illustrates the 2^1^*A*_*g*_^–^ – 1^1^*B*_*u*_^+^ level crossing while panel (b) illustrates the 1^1^*B*_*u*_^–^ – 1^1^*B*_*u*_^+^ level crossing. The absence of singlet-fission in lycopene under low-energy excitation is explained if a 2^1^*A*_*g*_^–^ – 1^1^*B*_*u*_^+^ level crossing occurs (i.e., case (a)). In this case, the 2^1^*A*_*g*_^–^ state is populated from the 1^1^*B*_*u*_^+^ state via Herzberg–Teller coupling and from which singlet fission is endothermic. However, high-energy excitation of a H-aggregate implies that a bright ^1^*B*_*u*_^+^ state lies higher in energy than the 1^1^*B*_*u*_^–^ state, allowing the latter to be populated (as discussed above and shown schematically in Figure 2). As already mentioned, once populated the 1^1^*B*_*u*_^–^ state can undergo exothermic intermonomer singlet fission, or slow (Herzberg–Teller allowed) internal conversion to the 2^1^*A*_*g*_^–^ state.

**Figure 3:**
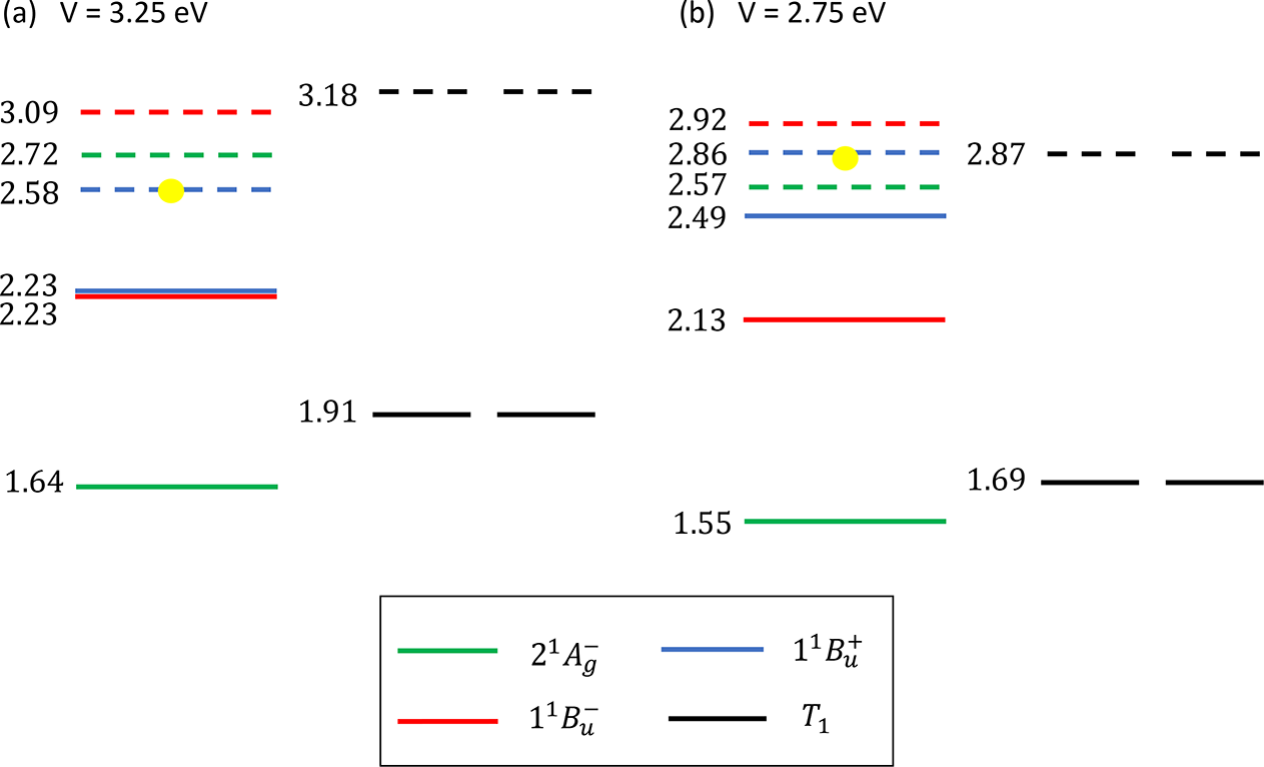
Vertical (dashed lines) and relaxed (solid lines) excitation energies (in eV) of the low-energy states of monomeric lycopene. These are obtained by DMRG calculations of the Hubbard-UV-Peierls model, as explained in SI. The *intra*monomer singlet states are on the left of each panel, while on the right of each panel is the energy level of two triplet states on separate monomers. In both cases, intermonomer singlet fission is (potentially) exothermic (endothermic) from the 1^1^*B*_*u*_^–^ (2^1^*A*_*g*_^–^) state. The yellow circle indicates the initially excited *monomeric* “bright” state. (a) The nearest-neighbor Coulomb repulsion, *V* = 3.25 eV; in this case there is level crossing between the 1^1^*B*_*u*_^+^ and 2^1^*A*_*g*_^–^ states. (b) *V* = 2.75 eV; in this case there is level crossing between the 1^1^*B*_*u*_^+^ and 1^1^*B*_*u*_^–^ states.

Assuming, now, that a singlet triplet-pair state has been formed (i.e., 1^1^*B*_*u*_^–^), we turn to discuss the singlet fission process. To simulate this process, we adopt the theory developed by Barford and Chambers to explain singlet fission in carotenoid dimers.^23^ The key assumptions of this theory are that singlet fission in carotenoid dimers occurs via one of the highly correlated dark states and that these dark states may be regarded as being composed entirely of a singlet triplet-pair. Therefore, the charge-transfer exciton component of the dark state^28^ is assumed to be a virtual component that acts to mediate the large intramonomer nearest neighbor triplet superexchange interaction,^23,37^
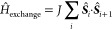
1Here, ***S*^** is the spin-1 (triplet) operator and *J* is the *inter*triplet exchange interaction. Thus, the intramonomer singlet and triplet triplet-pairs experience a nearest-neighbor *attraction*, 2*J* and *J*, respectively, while the quintet triplet-pair experiences a nearest-neighbor *repulsionJ*. As shown in ref (23), a value of *J* = 1.23 eV reproduces the intramonomer 1^5^*A*_*g*_^–^ – 2^1^*A*_*g*_^–^ energy gap of 0.4 eV for polyene chains.^29^

As explained more fully in ref (23), intra- and inter- lycopene monomer triplet transfer between adjacent ethylene dimers occurs by a superexchange mechanism via the (assumed) virtual charge-transfer exciton.^28^ Triplets hop between neighboring ethylene dimers on the same lycopene monomer with a transfer integral, *t*_intra_ = 0.88 eV,^23^ which results in a band of bound singlet triplet-pair states (the “2*A*_*g*_” family of states^29^), i.e., 2^1^*A*_*g*_^–^, 1^1^*B*_*u*_^–^, 3^1^*A*_*g*_^–^, ···. For convenience, these strongly interacting, intramonomer states are labeled ^1^|*TT*⟩. Higher in energy is a band of noninteracting intramonomer triplet-pair states.

Intermonomer triplet-pair states do not experience the strong intramonomer exchange interaction, and consequently (neglecting for the moment the dipolar interaction to be introduced shortly), the intermonomer triplet-pairs are noninteracting and space-separated. These pairs are labeled ^1^|*T*···*T*⟩, ^3^|*T*···*T*⟩ and ^5^|*T*···*T*⟩ for the singlet, triplet and quintet states, respectively.

Triplets hop between adjacent ethylene dimers on neighboring lycopene monomers with a transfer integral, *t*_inter_ = 0.0088 eV. This mechanism causes the intra- and inter monomer singlet triplet-pairs to hybridize to form the lowest energy bimolecular singlet state
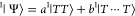
2

As is shown in ref (23), the mixing ratio *a*/*b* depends on the energy difference, Δ, between ^1^|*TT*⟩ and ^1^|*T*···*T*⟩, with an exothermic process implying that |*b*|^2^ > |*a*|^2^. As also shown in ref (23), a key emergent energy scale is Δ*E*_*QS*_, the exchange energy between the dimer singlet triplet-pair, ^1^|Ψ⟩, and the intermonomer quintet, ^5^|*T*···*T*⟩. If Δ*E*_*QS*_ ≪ *k*_*B*_*T* full singlet fission occurs and the population of the initial ^1^|*TT*⟩ becomes equally equilibrated between the singlet, triplet, and quintet intermonomer pairs, implying free triplets on separate monomers. Using the DMRG results shown in Figure 3a, in this simulation the exothermic driving energy (defined in Figure 2) is taken to be Δ = 0.32 eV. With this value of Δ, Δ*E*_*QS*_ = 4.9 × 10^–4^ eV. As explained in ref (23), Δ*E*_*QS*_ is the dimer triplet-pair binding energy, which is 3 orders of magnitude smaller than the intramonomer triplet-pair binding energy.

The final interaction to include in the triplet-pair Hamiltonian is the *intra*triplet dipolar (or zero-field-splitting) interaction. Assuming an axis of quantization along the principal dipolar axis, *Z*, this interaction reads^38^
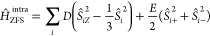
3where the sum is over both triplets in the pair, Ŝ are again the spin-1 operators, and  are the angular momentum shift operators. The first term couples the singlet triplet-pair with the *S*_*Z*_ = 0 component of the quintet triplet-pair, while the second term couples the singlet triplet-pair with the *S*_*Z*_ = ± 2 components of the quintet triplet-pair. Since *D* ∼ 10^–5^ eV is already small compared to other energy scales and *E* is typically 10 to 100 times smaller than *D*,^37,38^ the second term is neglected here.

In this work, we take the intermonomer triplet transfer integral, *t*_inter_, to be a parameter. In particular, the choice of *t*_inter_/*t*_intra_ = 0.01 predicts a ^1^|*TT*⟩ half-life of ca. 10 ps, which is consistent with experimental observations.^18^ As shown in the SI, this choice implies a separation between the lycopene monomers in the H-aggregate of ca. 3 Å, which is consistent with the experimental absorption blue-shift of 0.92 eV. Assuming a superexchange mechanism for triplet transfer,^23^ the value of the intermonomer triplet transfer integral, *t*_inter_, used here [see section 4 of the SI] is consistent with *ab initio* calculations of Ghosh et al.^21^

We now turn to discuss the dynamical simulation. The triplet-pair dynamics are determined by the quantum Liouville equation, which is computed in the eigenstate basis of the two-monomer triplet-pair Hamiltonian.^23^ Assuming the secular approximation, the quantum Liouville equation^39,40^ for the populations *P*_*a*_ ≡ ρ_*aa*_ is,
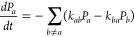
4while for the coherences it is,
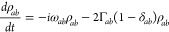
5The Bohr frequencies are ω_*ab*_ = (*E*_*a*_ – *E*_*b*_)/*ℏ*, while 2Γ_*ab*_ = (γ_*a*_ + γ_*b*_) and γ_*a*_ = *∑*_*b*≠*a*_*k*_*ab*_.

The inclusion of the ZFS interaction means that the energy eigenstates are not eigenstates of the total spin. In this simulation, we include both nonmagnetic and magnetic dephasing process. Thus, the thermal rates are a sum of the spin-conserving (SC) and spin-nonconserving (SNC) rates, i.e., *k*_*ab*_ = (*k*_*ab*_^SC^+*k*_*ab*_^SNC^), where *k*_*ab*_^SC^ and *k*_*ab*_^SNC^ are defined in the SI. Taking a reorganization energy of 0.05 eV,^6^ at 300 K the nonmagnetic dephasing time is calculated to be ca. 1 ps.^23^ We take the characteristic time for transverse (*S*_*z*_-conserving) magnetic dephasing, *T*_2_, to be ca. 10 ns. Assuming only transverse-spin dephasing and neglecting the spin-flip component of  means that only the *S*_*Z*_ = 0 components of the triplet and quintet triplet-pairs states are connected to the singlet triplet-pairs states.

The triplet-pair basis and the two-monomer triplet-pair Hamiltonian are described in section 3 of the SI; the parameters used in the simulation are listed in section 4 of the SI. The solution of eqn 4 and eqn 5 is described in ref (23).

**Figure 4:**
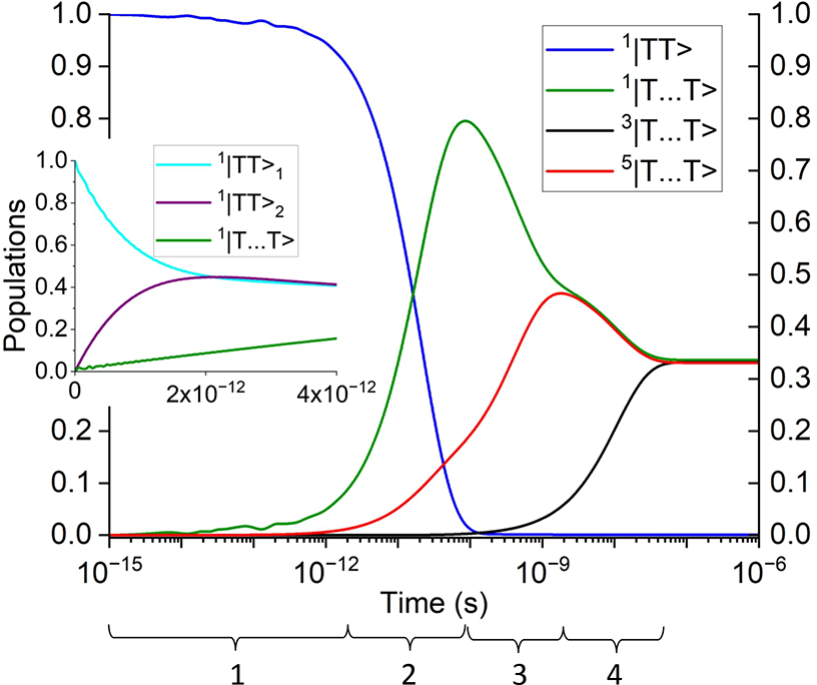
Populations as a function of time of the intramonomer singlet triplet-pairs, ^1^|*TT*⟩, and the intermonomer singlet, triplet and quintet triplet-pairs, ^1^|*T*···*T*⟩, ^3^|*T*···*T*⟩ and ^5^|*T*···*T*⟩. The four time regimes are discussed in the text. The inset shows the intramonomer singlet triplet-pair population on monomers 1 and 2 (i.e., ^1^|*TT*⟩_1_ and ^1^|*TT*⟩_2_), and the intermonomer singlet triplet-pair population, ^1^|*T*···*T*⟩. This figure should be compared to Figure 1.

We now discuss the results of the dynamical simulation. Taking as our initial state |Ψ⟩= ^1^|*TT*⟩ ≡|1^1^*B*_*u*_^–^⟩, the triplet-pair populations are illustrated in Figure 4. From this, we can identify four time regimes:

1. Up to ca. 2 ps there is very weak coherent dynamics between the intramonomer triplet pairs, ^1^|*TT*⟩, and the intermonomer triplet pair, ^1^|*T*···*T*⟩. As the system is off-resonance, the population is primarily ^1^|*TT*⟩. At 2 ps the populations of the intramonomer pairs on each lycopene monomer of the dimer are equal at 45%. This is represented as “1” in the experimental transient excited state absorption^18^ shown in Figure 1.
2. From ca. 2 ps to ca. 100 ps spin-conserving dephasing causes population transfer from ^1^|*TT*⟩ to ^1^|*T*···*T*⟩. Simultaneously, the ZFS interaction mixes the singlet and quintet intermonomer triplet-pair states. Since this process is over damped, there are no oscillations. By 100 ps the ^1^|*TT*⟩, ^1^|*T*···*T*⟩ and ^5^|*T*···*T*⟩ populations are 0%, 80% and 20%, respectively, and therefore singlet fission has occurred by this time.
3. From ca. 100 ps to ca. 2 ns the ZFS interaction continues to mix the singlet and quintet intermonomer triplet-pair states, becoming equal to 50% each at ca. 2 ns.

As ^1^|*T*···*T*⟩ and ^5^|*T*···*T*⟩ are spectroscopically indistinguishable, the time regimes 2 and 3 are represented as “2” in Figure 1.

4. Finally, after ca. 2 ns and within ca. 50 ns transverse spin-dephasing equilibrates ^1^|*T*···*T*⟩, ^3^|*T*···*T*⟩ and ^5^|*T*···*T*⟩ to a population of 1/3 each. Equivalently, this population corresponds to spin-uncorrelated, single triplets^23,41^ on separate monomers and is represented by “3” in Figure 1.

In summary, the proposed mechanism of singlet fission in lycopene H-aggregates is the following. Optical excitation of the aggregate excites a high-energy bright state (*S*_*n*_) that is partially delocalized over the aggregate. This state rapidly relaxes and localizes onto a single lycopene monomer, populating an intermediate singlet triplet-pair state. This is the 1^1^*B*_*u*_^–^ state, often labeled *S*_1_^*^. As explained in this Letter, this state is the second member of the strongly correlated “2*A*_*g*_” family of states. It is the strongly bound, intramonomer triplet-pair state, also labeled ^1^|*TT*⟩, whose population dynamics are illustrated in Figure 4. Internal conversion from the 1^1^*B*_*u*_^–^ state to the 2^1^*A*_*g*_^–^ state is assumed to be slow because it is symmetry forbidden. Next, the 1^1^*B*_*u*_^–^ state undergoes “fission” into noninteracting, spin-correlated triplet pairs on separate monomers, labeled ^1^|*T*···*T*⟩. Because fission from the 1^1^*B*_*u*_^–^ state is quite exothermic, within ca. 100 ps there is almost complete population transfer from ^1^|*TT*⟩ to ^1^|*T*···*T*⟩. Following the population of ^1^|*T*···*T*⟩, the ZFS interaction mixes ^1^|*T*···*T*⟩ and ^5^|*T*···*T*⟩. Finally, hyperfine interactions mix ^1^|*T*···*T*⟩, ^3^|*T*···*T*⟩ and ^5^|*T*···*T*⟩. Since Δ*E*_*QS*_/*k*_*B*_*T* ≃ 0.02 (and we consider only transverse dephasing), the populations are equal. This final mixed state corresponds to spin-uncorrelated, single triplets on separate monomers.^23,41^

This Letter has focused on singlet fission in lycopene H-aggregates. However, other carotenoid H-aggregates also exhibit singlet fission with a photophysical behavior semiquantitatively similar to that described here. For example, Quaranta and co-workers^19^ investigated singlet fission in lutein and violaxanthin H-aggregates. In common with lycopene, these carotenoids also possess *C*_2*h*_ symmetry. Following photoexcitation of the aggregate, Quaranta and co-workers^19^ propose an intermediate state that participates in singlet fission, which they nominated as a vibrationally hot *S*_1_ state. In light of the work described here, however, we think that this state is more likely to be a distinct (albeit a related) electronic state, namely, the 1^1^*B*_*u*_^–^ state.

In principle, the model proposed here can also be applied to other carotenoid aggregate systems. For example, Musser and co-workers^16^ investigated singlet fission in a wide-range of astaxanthin aggregates, with the aggregate absorption ranging from 1.9 to 3.1 eV. As these excitation energies overlap or are higher than the corresponding intramonomer 1^1^*B*_*u*_^–^ energy,^30^ population of and subsequent single fission from this state is possible.

In conclusion, this Letter describes a theory of singlet fission in lycopene H-aggregates that is in semiquantitative agreement with experimental observations.^18^ In particular, the theory assumes that singlet fission in carotenoid systems occurs via an intermediate intramonomer singlet triplet-pair state (i.e., 1^1^*B*_*u*_^–^), which facilitates exothermic intermonomer singlet fission. This state is populated via the excitation of a higher energy H-aggregate bright state. In contrast, singlet fission in polyacenes occurs directly from the intramolecular bright state. Thus, the participation of an intramolecular triplet-pair state in carotenoid singlet fission implies that this mechanism is quite different from that of polyacenes.

## Supporting Information Available

The Supporting Information contains the following sections: 1. Hubbard-UV-Peierls Hamiltonian, 2. Excited State Absorption, 3. Triplet-Pair Basis and Hamiltonian, 4. Model Parameters, 5. Computation of Interstate Rates, and 6. Intermonomer Triplet-Pair Coupling (PDF)
