# Supplementary material for: Singlet Fission in Lycopene H-Aggregates
Source: J Phys Chem Lett. 2023 Oct 27;14(44):9842–7. doi: 10.1021/acs.jpclett.3c02435 (PMC10641873; doi:10.1021/acs.jpclett.3c02435)
Supplement: Supplementary file 1 — jz3c02435_si_001.pdf [file jz3c02435_si_001.pdf]

Singlet Fission in Lycopene H-Aggregates
Supporting Information

William Barford*

Department of Chemistry, Physical and Theoretical Chemistry Laboratory, University of Oxford, Oxford, OX1 3QZ, United Kingdom

E-mail: william.barford@chem.ox.ac.uk

Table of Contents

1. Hubbard-UV-Peierls Hamiltonian
2. Excited State Absorption
3. Triplet-Pair Basis and Hamiltonian
4. Model Parameters
5. Computation of Interstate Rates
6. Intermonomer Triplet-Pair Coupling

1. Hubbard-UV-Peierls Hamiltonian

The DMRG calculations[1,2] of the electronic states of lycopene were performed using the Hubbard-UV-Peierls Hamiltonian. This Hamiltonian has three components.

The purely electronic Hubbard-UV Hamiltonian is

$$\hat{H}_{\text{UV}} = -2\sum_{n=1}^{N-1}\beta_n\hat{T}_n + U\sum_{n=1}^{N}\big(\hat{N}_{n\uparrow}-\frac{1}{2}\big)\big(\hat{N}_{n\downarrow}-\frac{1}{2}\big) + \frac{1}{2}\sum_{n=1}^{N-1}V\big(\hat{N}_n-1\big)\big(\hat{N}_{n+1}-1\big), \tag{1}$$

which contains a nearest neighbor electron transfer term, β_n, and onsite and nearest neighbor Coulomb interactions, U and V, respectively. $\hat{T}_n = \frac{1}{2}\sum_\sigma\left(c^\dagger_{n,\sigma}c_{n+1,\sigma} + c^\dagger_{n+1,\sigma}c_{n,\sigma}\right)$ is the bond order operator, $\hat{N}_n$ is the number operator and N ($= 22$ for lycopene) is the number of conjugated carbon-atoms ($N/2$ is the number of double bonds).

The electrons couple to the nuclei via changes in the C-C bond length (which changes the effective electron transfer integral)

$$\hat{H}_{\text{e-n}} = 2\alpha\sum_{n=1}^{N-1}\left(u_{n+1}-u_n\right)\hat{T}_n, \tag{2}$$

where α is the electron-nuclear coupling parameter and u_n is the displacement of nucleus n from its undistorted position.

Finally, the nuclear potential energy is described by

$$\hat{H}_{\text{elastic}} = \frac{K}{2}\sum_{n=1}^{N-1}\left(u_{n+1}-u_n\right)^2, \tag{3}$$

where K is the nuclear spring constant.

The Hubbard-UV-Peierls Hamiltonian is defined as

$$\hat{H}_{\text{UVP}} = \hat{H}_{\text{UV}} + \hat{H}_{\text{e-n}} + \hat{H}_{\text{elastic}}. \tag{4}$$

This Hamiltonian is invariant under both a two-fold proper rotation (i.e., a C_2 operation)

and a particle-hole transformation (i.e., $(\hat{N}-1) \to -(\hat{N}-1)$), and so its eigenstates are labeled either $A_g^{\pm}$ or $B_u^{\pm}$.

The parameters are the same as used in previous work,[3,4] i.e., $\beta = 2.4$ eV, $U = 7.25$ eV, $K = 46$ eV $\AA^{-2}$ and $\alpha = 4.6$ eV $\AA^{-1}$. As described in the main paper, in order to model relative $2^1A_g^-/1^1B_u^+$ energies we make two choices for V, i.e., $V = 2.75$ eV or $V = 3.25$ eV.

2. Excited State Absorption

Using DMRG,[1] the excited state absorption is calculated from the Hubbard-UV-Peierls Hamiltonian using the parameter set for a $2^1A_g^-/1^1B_u^+$ energy level crossover, i.e., $V = 3.25$ eV. This is shown in Fig. 1.

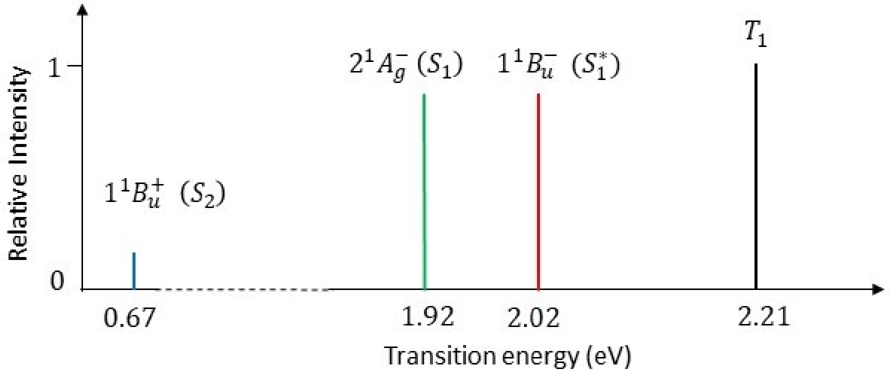

Figure 1: The DMRG calculated excited state absorption from a lycopene monomer, showing the red-shift of the 'triplet-pair' state absorption relative to a free triplet. As shown in refs,[4,5] this absorption arises from the charge-transfer exciton component of these states. Compare to the experimental results[6] shown in Fig. 1 of the main paper.

3. Triplet-Pair Basis and Hamiltonian

The initial singlet triplet-pair state, $^1|TT\rangle$, is[7]

$$^1|\mathrm{TT}\rangle = \sum_{ij \in \times = 1} \Phi_{ij}{}^1|i,j\rangle, \tag{5}$$

where the singlet triplet-pair basis state is

$$^1|i,j\rangle = \frac{1}{\sqrt{3}} \left(|1;i\rangle| - 1;j\rangle - |0;i\rangle|0;j\rangle + | - 1;i\rangle|1;j\rangle \right), \tag{6}$$

$|S_Z;i\rangle$ is a triplet on ethylene dimer i with spin projection S_Z, Φ_{ij} is the lowest eigenstate of the one-monomer Hamiltonian (defined below) with B_u symmetry, and $ij \in \times = 1$ in eqn (5) implies that dimers i and j are on monomer 1.

As described in the main paper, triplets on the same lycopene monomer experience the nearest-neighbor exchange interaction,[8,9]

$$\hat{H}_{\text{exchange}} = J \sum_i \hat{\mathbf{S}}_i \cdot \hat{\mathbf{S}}_{i+1}, \tag{7}$$

where $\hat{\mathbf{S}}$ is the spin-1 (triplet) operator and J is the *inter*triplet exchange interaction. Thus, the intramonomer singlet and triplet triplet-pairs experience a nearest-neighbor attraction, $+2J$ and $+J$, respectively, while the quintet triplet-pair experiences a nearest-neighbor repulsion $-J$.

Triplets also experience the *intra*triplet dipolar (or zero-field-splitting) interaction,

$$\hat{H}^{\text{intra}}_{\text{ZFS}} = \sum_i D \left(\hat{S}^2_{iZ} - \frac{1}{3} \hat{S}^2_i \right), \tag{8}$$

where only the S_Z conserving component is retained and the sum is over both triplets.

In this work we investigate the role of transverse spin-dephasing and the component of the ZFS-Hamiltonian which connects the $S_z = 0$ components of each total spin. The $S_z = 0$

components of the triplet and quintet triplet-pair bases are,

$$^{3}|i,j\rangle = \frac{1}{\sqrt{2}}\left(|1;i\rangle|-1;j\rangle - |-1;i\rangle|1;j\rangle\right), \tag{9}$$

and

$$^{5}|i,j\rangle = \frac{1}{\sqrt{6}}\left(|1;i\rangle|-1;j\rangle + 2|0;i\rangle|0;j\rangle + |-1;i\rangle|1;j\rangle\right), \tag{10}$$

respectively.

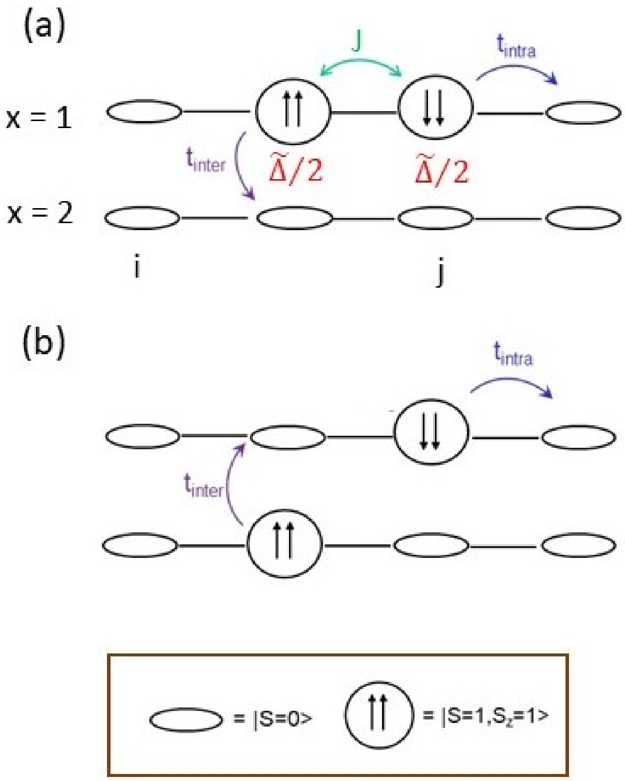

Figure 2: A schematic illustration of a carotenoid dimer. (a) with a triplet-pair on monomer $\times = 1$, with triplets on ethylene dimers i and j. Monomer $\times = 2$ is in its ground state. This represents $\hat{H}_{\text{exchange}} + \hat{H}^{\times=1,2}_{\text{single}}$. (b) represents $\hat{H}_{\text{double}} + \hat{H}_{\text{inter}}$. t_{intra} and t_{inter} are the hopping matrix elements between neighboring intramonomer and intermonomer ethylene dimers, respectively.

The triplets hop between neighboring dimers on the same monomer (see Fig. 2(a)),

described by

$$\hat{H}_{\text{single}}^{\times=1,2} = \tilde{\Delta} \sum_{i,j>i\in\times} |i,j\rangle\langle i,j| + t_{\text{intra}} \sum_{i\neq j\in\times} \left(|i\pm 1,j\rangle\langle i,j| + \text{H.C.}\right), \tag{11}$$

Defining BE as the *intra*monomer singlet triplet-pair binding energy with respect to two free triplets on separate monomers,[9] then $\Delta = (\tilde{\Delta} - \text{BE})$ is the exothermic driving energy of the intramonomer triplet-pair relative to the intermonomer free triplets. Δ is defined in Fig. 2 of the main paper for $1^1B_u^-$ state. Triplets on separate monomers hop between neighboring dimers on the same monomer (see Fig. 2(b)), described by

$$\hat{H}_{\text{double}} = t_{\text{intra}} \sum_{i\in\times=1} \sum_{j\in\times=2} \left[\left(|i\pm 1,j\rangle\langle i,j| + \text{H.C.}\right) + \left(|i,j\pm 1\rangle\langle i,j| + \text{H.C.}\right)\right], \tag{12}$$

and between adjacent dimers on neighboring monomers (see Fig. 2(b)), described by

$$\hat{H}_{\text{inter}} = t_{\text{inter}} \sum_{\times=1}^{2} \sum_{i_\times} \sum_{j_\times>i_\times} \left[\left(|i_\times,j_\times\rangle\langle i_{\bar{\times}},j_\times| + \text{H.C.}\right) + \left(|i_\times,j_\times\rangle\langle i_\times,j_{\bar{\times}}| + \text{H.C.}\right)\right]. \tag{13}$$

Intra- and inter- lycopene monomer triplet transfer occurs by a superexchange mechanism via the (assumed) virtual charge-transfer exciton,[7] as explained more fully in ref.[9]

4. Model Parameters

Table 1 lists the parameters used in the two-monomer triplet-pair Hamiltonian described in Section 2, as well as the parameters used in the quantum Liouville equation.

5. Computation of Interstate Rates

The inclusion of the ZFS interaction into the two-monomer triplet-pair Hamiltonian means that the energy eigenstates are not eigenstates of total spin. In the simulation we include

Table 1: Values of input and derived parameters.

Parameter	Value
Intramonomer triplet exchange interaction,[9] J	1.23 eV
Intramonomer triplet transfer integral,[7] t_{intra}	0.88 eV
Intermonomer triplet transfer integral, t_{inter}	0.0088 eV
Exothermic driving energy, Δ	0.32 eV
Intratriplet dipolar interaction, D	10^{-5} eV
Reorganization energy, λ	0.05 eV
Spectral function cut-off frequency, ω_0	0.2 eV
k_BT	26 meV
Spin-dephasing factor, γ	10^{-4}
Derived transverse magnetic dephasing time, T_2	$\sim$ 10 ns
Derived nonmagnetic dephasing time	$\sim$ 1 ps
Derived two-monomer quintet-singlet exchange energy, ΔE_{QS}	4.9×10^{-4} eV

both nonmagnetic and magnetic dephasing processes. To account for this the thermal rates that appear in quantum Liouville equation are defined as the sum of the spin-conserving (SC) and spin-nonconserving (SNC) rates, i.e.,

$$k_{ab} = k_{ab}^{\text{SC}} + k_{ab}^{\text{SNC}}. \tag{14}$$

Defining the Bohr frequencies as $\omega_{ab} = (E_a - E_b)/\hbar$ and taking $\omega_{ab} \geq 0$, the spin-conserving thermal rates are,[10,11]

$$k_{ab}^{\text{SC}} = \left(\frac{2\lambda}{\hbar}\right) J(\omega_{ab})(n(\omega_{ab}) + 1)C_{ab}^{\text{SC}} \tag{15}$$

and

$$k_{ba}^{\text{SC}} = \left(\frac{2\lambda}{\hbar}\right) J(\omega_{ab})n(\omega_{ab})C_{ab}^{\text{SC}}, \tag{16}$$

where $n(\omega) = (\exp \beta\hbar\omega - 1)^{-1}$ is the Bose distribution function, $J(\omega) = \omega\omega_0/(\omega^2 + \omega_0^2)$ is the (dimensionless) Debye-spectral function, ω_0 is the cut-off frequency and λ is the bath reorganization energy. The parameters are listed in Table 1.

The spin-conserving overlap factors are,

$$C_{ab}^{\mathrm{SC}} = 2\sum_{m} S_{ma}^2 S_{mb}^2. \tag{17}$$

Here, a and b label energy eigenstates of the two-monomer Hamiltonian, whereas m labels a real-space basis state of the triplet-pair states.[9] In particular, m encodes the dimer locations of each triplet in the pair, as well as the total spin of the pair. $\mathbf{S}$ is the matrix whose columns are the eigenvectors of the two-monomer Hamiltonian represented in the real-space basis. Thus, $S_{ma}^2 = P_{ma}$ is the probability that the ath energy eigenstate occupies the mth real-space basis state.

Similarly, the spin-nonconserving thermal rates are,

$$k_{ab}^{\mathrm{SNC}} = \gamma\left(\frac{2\lambda}{\hbar}\right) J(\omega_{ab})(n(\omega_{ab})+1)C_{ab}^{\mathrm{SNC}} \tag{18}$$

and

$$k_{ba}^{\mathrm{SNC}} = \gamma\left(\frac{2\lambda}{\hbar}\right) J(\omega_{ab})n(\omega_{ab})C_{ab}^{\mathrm{SNC}}, \tag{19}$$

where γ is a factor to take into account weaker transverse spin-dephasing than spin-conserving dephasing. We take $\gamma = 10^{-4}$, which at 300 K implies $T_2 \sim 10$ ns.

The spin-nonconserving overlap factors are,

$$C_{ab}^{\mathrm{SNC}} = \sum_{m} f_{m\bar{m}}\left(S_{ma}^2 S_{\bar{m}b}^2 + S_{\bar{m}a}^2 S_{mb}^2\right), \tag{20}$$

where the label $\bar{m}$ refers to the same triplet-pair dimers as m, but corresponds to a different spin-eigenstate, i.e., singlet, triplet or quintet. In addition, $f_{m\bar{m}} = 2/3$ for singlet-triplet transitions, $f_{m\bar{m}} = 1/3$ for triplet-quintet transitions, and $f_{m\bar{m}} = 0$ for singlet-quintet transitions.[9]

6. Intermonomer Triplet-Pair Coupling

The simulations of singlet fission in this work took the intermonomer triplet transfer integral, t_{inter}, to be a parameter. In particular, the choice of $t_{\text{inter}}/t_{\text{intra}} = 0.01$ results in a half-life of the intramonomer singlet triplet-pair, $^1|TT\rangle$, to be ca. 10 ps. This value is consistent with the experimental observations of Kundu and Dasgupta[6] (see Fig. 1 of the main paper). Indeed, the $^1|TT\rangle$ half-life is rather sensitive to $t_{\text{inter}}/t_{\text{intra}}$: values of $t_{\text{inter}}/t_{\text{intra}} = 0.01$, 0.001 and 0.0001 predict half-lives of 10 ps, 1 ns and 100 ns, respectively.

As explained in ref,[9] both intra and inter monomer triplet transfer is assumed to be a superexchange process, mediated by a virtual charge-transfer exciton. Thus, $t \propto \beta^2$, where β is the p_z-orbital resonance integral. We therefore deduce that $\beta_{\text{inter}}/\beta_{\text{intra}} = 0.1$. Using the Mulliken expression[12] for β, i.e.,

$$\beta = (10.6 \text{ eV}) \times \exp(-r\xi) \left(1 + r\xi + \frac{2}{5}(r\xi)^2 + \frac{1}{15}(r\xi)^3\right), \tag{21}$$

where r is in Å and $\xi = 3.07$ Å^{-1}, we can now estimate the intermolecular separation. Taking the single bond length $r_{\text{single}} = 1.45$ Å implies that $\beta_{\text{intra}} = 2.4$ eV and thus $\beta_{\text{inter}} = 0.24$ eV. Again, using eqn (21), we find that the intermonomer separation is thus predicted to be 2.9Å.

As we now show, a separation of ca. 3 Å between the lycopene monomers in the H-aggregate is consistent with a blue shift of 0.92 eV, as measured in ref.[6] According to the line-dipole theory of exciton transfer integrals,[13] the exciton transfer integral, J, between two conjugated molecules of length L and separation r is

$$J = \left(\frac{\mu^2}{4\pi\epsilon_0 r^3}\right)\left(\frac{2}{(L/r)^2}\right)\left(1 - \frac{1}{\sqrt{1+(L/r)^2}}\right), \tag{22}$$

where μ is the transition dipole moment.

According to DMRG calculations,[1] $\mu = 5.71 \times 10^{-29}$ Cm for a lycopene monomer of

22 conjugated C-atoms. The measured blue-shift in a H-aggregate is approximately zJ, where z is the number of nearest neighbors to which each monomer is dipole coupled. Using $zJ = 0.92$ eV and $r = 2.9$ Å, we find that $z = 4.6$. This result seems very reasonable and places confidence that, as well as being empirically justified, our parameter choice of $t_{\text{inter}}/t_{\text{intra}} = 0.01$ is physically realistic.

References

(1) Barford, W.; Bursill, R. J.; Lavrentiev, M. Y. Density-Matrix Renormalization-Group Calculations of Excited States of Linear Polyenes. *Phys. Rev. B* **2001**, *63*, 195108.

(2) Manawadu, D.; Valentine, D. T.; Barford, W. Dynamical Simulations of Carotenoid Photoexcited States Using Density Matrix Renormalization Group Techniques. *J. Phys. Chem. A* **2023**, *127*, 3714–3727.

(3) Manawadu, D.; Valentine, D. J.; Marcus, M.; Barford, W. Singlet Triplet-Pair Production and Possible Singlet-Fission in Carotenoids. *J. Phys. Chem. Lett.* **2022**, *13*, 1344–1349.

(4) Manawadu, D.; Georges, T. N.; Barford, W. Photoexcited State Dynamics and Singlet Fission in Carotenoids. *J. Phys. Chem. A* **2023**, *127*, 1342–1352.

(5) Valentine, D. J.; Manawadu, D.; Barford, W. Higher-Energy Triplet-Pair States in Polyenes and their Role in Intramolecular Singlet Fission. *Phys. Rev. B* **2020**, *102*, 125107.

(6) Kundu, A.; Dasgupta, J. Photogeneration of Long-Lived Triplet States through Singlet Fission in Lycopene H-Aggregates. *J. Phys. Chem. Lett.* **2021**, *12*, 1468–1474.

(7) Barford, W. Theory of the Dark State of Polyenes and Carotenoids. *Phys. Rev. B* **2022**, *106*, 35201.

(8) Kollmar, C. Electronic-Structure of Diradical and Dicarbene Intermediates in Short-Chain Polydiacetylene Oligomers. *J. Chem. Phys.* **1993**, *98*, 7210–7228.

(9) Barford, W.; Chambers, C. A. Theory of Singlet Fission in Carotenoid Dimers. *J. Chem. Phys.* **2023**, *159*, 084116.

(10) Nitzan, A. *Chemical Dynamics in Condensed Phases: Relaxation, Transfer and Reactions in Condensed Molecular Systems*; Oxford University Press: Oxford, 2006.

(11) May, V.; Kühn, O. *Charge and Energy Transfer Dynamics in Molecular Systems*; Wiley-VCH: Weinheim, 2011.

(12) Mulliken, R. S.; Rieke, C. A.; Orloff, D.; Orloff, H. Overlap Integrals and Chemical Binding. *J. Chem. Phys.* **1949**, *17*, 510.

(13) Barford, W. Exciton transfer integrals between polymer chains. *J. Chem. Phys.* **2007**, *126*, 134905.
